# Mechanism study on the treatment of ulcerative colitis by Gegen Qinlian nano-preparation through promoting M2 macrophage polarization

**DOI:** 10.3389/fmolb.2025.1580874

**Published:** 2025-04-25

**Authors:** Jilei Li, Jiarui Cao, Zhenyu Zhang, Sizhe Wang, Meng Zhu, Lili Yang, Wenhui Ouyang, Chunzheng Ma

**Affiliations:** ^1^ Henan Province Hospital of TCM, Zhengzhou (The Second Affiliated Hospital of Henan University of Chinese Medicine), Zhengzhou, Henan, China; ^2^ Henan University of Chinese Medicine (The Second Clinical Medical College of Henan University of Chinese Medicine), Zhengzhou, Henan, China; ^3^ Kaifeng Central Hospital, Kaifeng, Henan, China

**Keywords:** ulcerative colitis, Gegen Qinlian nano-preparation, nanoparticle-targeted traditional Chinese medicine, macrophages, polarization

## Abstract

**Objective:**

To address the core pathological features of intestinal barrier disruption and immune imbalance in ulcerative colitis (UC), we developed a nano-targeted formulation (GGQL nano-preparation) based on berberine, puerarin, baicalin, and glycyrrhizin by combining traditional Chinese medicine (TCM) and nanotechnology in this study. We aimed to investigate whether GGQL nano-preparation could promote M2 macrophage polarization, correct intestinal inflammation, and treat UC.

**Methods:**

We used databases to identify M2 macrophage-related gene targets for GGQL nano-preparation in UC. Protein-protein interaction networks, topological analysis, and GO/KEGG enrichment analyses revealed GGQL nano-preparation‘s potential regulation of macrophage polarization via a specific pathway. We validated this using a dextran sulfate sodium (DSS)-induced UC model in C57BL/6 mice. Parameters assessed included the disease activity index (DAI), colon length, colitis macroscopic damage index (CMDI), spleen index, and pathological changes (via HE staining). Immunohistochemistry detected AMPK-PPAR axis factor changes to determine GGQL nano-preparation’s impact on M2 macrophage polarization and intestinal inflammation.

**Results:**

Our analyses suggested the GGQL nano-preparation reduced the DAI, enhanced colon length, improved CMDI scores, and mitigated splenic inflammation. HE staining showed GGQL nano-preparation alleviated inflammation in the spleen, lungs, and colon. Immunohistochemical findings indicated GGQL nano-preparation upregulated AMPK, PPAR, and SIRT1 expression. Mechanistically, GGQL nano-preparation promoted M2 macrophage polarization through the AMPK-PPARγ axis, achieving therapeutic objectives for UC.

**Conclusion:**

The GGQL nano-preparation effectively promotes M2 macrophage polarization via the AMPK-PPARγ axis, treating UC.

## 1 Introduction

Ulcerative Colitis (UC) is a chronic inflammatory disorder of indeterminate etiology that predominantly involves the mucosal and submucosal layers of the colon and rectum. Clinically, it is distinguished by enduring symptoms, including abdominal pain, diarrhea, mucoid stools, and hematochezia (Ordas et al., 2012; Segal et al., 2021). Patients with UC who persist in a chronic inflammatory condition face an elevated risk of developing colorectal cancer and other gastrointestinal malignancies. The prevalence of UC is increasing in developing nations, notably in China and other parts of Asia. Despite substantial progress in research, the fundamental mechanisms underlying UC remain poorly understood. These mechanisms may be affected by a multifaceted interplay of factors, including dietary influences (Levine et al., 2020), genetics, environmental triggers, and immune dysregulation (Shen et al., 2021). Furthermore, the aberrant expression of specific genes can significantly contribute to the pathogenesis of UC. A recent study, for instance, identified the cyclic RNA circNlgn as a pivotal factor in the promotion of colitis. The protein isoform encoded by circNlgn, Nlgn173, facilitates the phosphorylation of tyrosine-53 on nuclear actin upon its nuclear entry. This modification impairs the interaction between nuclear actin and the Arp2/3 complex, resulting in a reduction of filamentous actin levels. Consequently, this disruption in actin dynamics perturbs the balance between apoptosis and cellular proliferation, thereby exacerbating the onset and progression of colitis (Du WW et al., 2024).

In addition to the above mentioned predisposing factors for the development of UC, gut barrier disruption plays a crucial role in the onset and progression of UC, with gut immune dysfunction being the key factor in damaging the gut barrier. Currently, most views suggest that excessive immune responses in the intestine are closely linked to UC. Excessive immune responses in the gut cannot be separated from imbalances in intestinal macrophage polarization (Kaminsky et al., 2021). Macrophages are divided into M1 and M2 subtypes (Xu Z. et al., 2022), and the imbalance in M1/M2 macrophage polarization exacerbates intestinal inflammation (Wang et al., 2023). M1-type macrophages are critical factors in initiating inflammatory responses and produce inflammatory cytokines such as interleukin (IL)-1, IL-12, and tumor necrosis factor-α (TNF-α) (Nakase et al., 2022), and also release reactive oxygen species (ROS), nitric oxide, etc., which in turn damage intestinal mucosa. M2-type macrophages play a key role in immune regulation (Locati et al., 2020), synthesizing IL-10, transforming growth factor-β (TGF-β), and fibroblast growth factors to promote immune suppressive reactions and perform tissue repair functions (Dharmasiri et al., 2021), and inhibiting excessive immune responses, promoting tissue repair and fibrosis (Zhu et al., 2020; Xu et al., 2024; Barberio et al., 2021) The occurrence of UC is often associated with the over-polarization of MI macrophages. Therefore, by balancing macrophage M1/M2 polarization and reconstructing the immune network, UC can be effectively ameliorated, which is a new direction for the treatment of UC. The contemporary Western pharmacological treatment for UC primarily comprises amino salicylate agents (5-ASA) (Barberio et al., 2021), glucocorticoids, immunosuppressants (Gordon et al., 2022), biologics (D'Haens et al., 2023), and small molecule agents (Wang et al., 2021). For patients with mild to moderately active UC, the main approach to induce remission is through the use of 5-ASA; however, not all patients respond effectively to this treatment. In cases where UC is refractory to 5-ASA, treatment typically transitions to corticosteroids, probiotics, immunotherapy, and fecal microbiota transplantation (FMT) (Biemans et al., 2020). Nevertheless, the long-term administration of these medications can lead to severe adverse effects, including endocrinological disorders, hypertension, and lymphoma (Higashiyama and Hokari, 2023).

Ancient Chinese medical texts, such as the “Synopsis of the Golden Chamber” and the “Simple Questions on Qi Diseases,” have documented numerous Chinese herbal formulations that are efficacious in treating UC and alleviating its symptoms. Traditional Chinese medicine (TCM) approaches UC treatment based on syndrome differentiation, employing therapeutic strategies such as clearing heat and promoting diuresis, detoxifying by clearing heat, invigorating the spleen and replenishing qi, warming the kidney, and strengthening the spleen. Research suggests that TCM can effectively enhance various barriers to the intestinal mucosa by mechanisms such as restoring tight junction proteins, inhibiting inflammatory cell infiltration, regulating gut microbiota and their metabolites, and inducing mucin production (Cui et al., 2021; Shi et al., 2021; Zhao et al., 2023; Xu et al., 2023). For example, Gingerenone A (GA) directly interacts with IL-17RA protein through pull-down, surface plasmon resonance analysis, and molecular dynamics simulation to inhibit inflammatory signaling activation (Liang et al., 2024). GGQL decoration is a commonly used formula for the treatment of UC and has shown significant clinical efficacy. However, the low content of active components and poor drug utilization in GGQL decoration limit its therapeutic effects. To improve therapeutic efficacy, we have identified four anti-UC components: berberine, puerarin, baicalin, and glycyrrhizin. These components have been incorporated into a nanometric targeted drug delivery system to create a novel TCM formulation. This nanomedicine is specifically designed to address the pathological characteristics of high ROS levels and low pH in the area of colon mucosal injury. It utilizes a hyaluronic acid-chitosan dual-responsive copolymer as the delivery vehicle. Through ultrasonic dispersion technology, the four anti-UC components from GGQL nano-preparation, including puerarin and berberine, are effectively loaded onto the carrier. The nanomedicine remains stable within the healthy colon but, upon reaching the lesion site, the carrier responds to the ROS/pH environment, leading to rapid cleavage of chemical bonds and precise drug release. This mechanism significantly enhances drug concentration at the lesion site. The formulation offers several advantages, including targeted release to improve bioavailability, a viscous gel state to prolong local retention and form a protective membrane, and effective blockade of pathogenic microorganism invasion, thereby reducing the risk of secondary infections. Currently, many studies have confirmed that TCM can AMP-activated protein kinase (AMPK)-peroxisome proliferator-activated receptor gamma (PPARγ) axis regulating macrophage polarization and the expression and secretion of related inflammatory factors, such as IL-10, IL-1β, and IL-6, thereby reducing intestinal inflammation (Xu N. et al., 2022; Ye et al., 2020). Therefore, our study is based on the AMPK-PPARγ axis, using intestinal immune dysfunction as the entry point, to investigate the relationship between macrophage polarization imbalance and UC (UC). Additionally, we employ modern scientific and technological methods to explore the potential mechanism of the Gegen Qinlian formula component, specifically its nanotargeted Chinese medicine (hereinafter referred to as GGQL), in regulating macrophage polarization in UC. This research aims to provide valuable references for the prevention and treatment of UC.

## 2 Methods

### 2.1 GGQL component target acquisition

We utilized the PubChem database to procure the PubChem Compound Identifier (CID) and Canonical Simplified Molecular Input Line Entry System (SMILES) representations for the components. Additionally, we employed the SwissTargetPrediction database (http://www.swisstargetprediction.ch/, with a probability threshold greater than 0) and the BATMAN 2.0 database (http://bionet.ncpsb.org.cn/batman-tcm/, with a score cutoff exceeding 0.84) to search component targets. Furthermore, targets were acquired from the Chinese Herbal Medicine Database (http://herb.ac.cn/). All retrieved data from these databases were compiled and duplicates were eliminated to ascertain the targets associated with GGQL.

### 2.2 UC differential gene acquisition

Utilizing the keyword “UC, *Homo sapiens*,” gene expression data (GSE92415) were obtained from the Gene Expression Omnibus (GEO) database (http://www.ncbi.nlm.nih.gov/geo/). This dataset comprises 87 UC patients and 21 healthy controls. Differentially expressed genes (DEGs) in GSE92415 were identified using the “limma” R package. The criteria for DEG screening were set at an absolute log_2_ fold change greater than 0.5 and an adjusted p-value less than 0.05. Heatmaps and volcano plots of the DEGs were constructed using the “pheatmap” and “ggplot2” R packages, respectively.

### 2.3 CIBERSORT immune infiltration analysis

To determine the relative abundance of immune cells in the samples, we employed CIBERSORT to analyze the UC expression data. Following the guidelines of CIBERSORT L22, the mRNA expression matrix was analyzed using the CIBERSORT R script obtained from the CIBERSORT website. The Wilcoxon rank-sum test was utilized to evaluate differences in the proportions of immune cells between groups, with statistical significance set at p < 0.05.

### 2.4 WGCNA analysis

Based on the immune infiltration phenotype, specifically focusing on M2 macrophages, a weighted gene co-expression network analysis (WGCNA) was performed using the “WGCNA” R package. The analysis involved several steps: first of all, hierarchical clustering was conducted on the study samples to identify and exclude outliers. Apart from that, a scale-free network was constructed, and the soft threshold β = 14 was selected based on the pickSoftThreshold function, achieving a scale-free topology fit index (R2) of 0.798. Then, an adjacency matrix was established and transformed into a topological overlap matrix (TOM). Gene dendrograms and module colors were generated based on different connectivity degrees. Finally, the correlation between each module and the differential samples was calculated to identify relevant gene modules.

### 2.5 Translation of GGQL treatment for UC: M2 macrophage target acquisition

Using the UniProt database, we standardized the disease targets and potential targets of drug components to Gene Symbols. Through a Venn diagram (http://www.bioinformatics.com.cn), we selected the intersection of GGQL, UC-M2, and UC-DEGs.

### 2.6 Protein interaction network construction

We imported the intersection target dataset into the STRING database (https://string-db.org/), selecting the species as *H. sapiens* and setting the minimum required interaction score to 0.4. We downloaded the “string_interactions_short.tsv” file. This file was then imported into Cytoscape 3.8.2 for network visualization. The text size, color depth, and node size were set according to the Degree value; larger Degree values resulted in larger text, darker colors, and larger nodes, indicating higher importance of the node in the protein interaction network. We used the CytoNCA plugin for network topology analysis, calculating node Degree Centrality (DC), Betweenness Centrality (BC), Closeness Centrality (CC), Eigenvector Centrality (EC), Local average connectivity-based method (LAC), and Network Centrality (NC). We employed the Cytohubba plugin to compute core targets of the intersection genes using four algorithms as the core. Additionally, we used the MCODE plugin for cluster analysis of the protein interaction network.

### 2.7 GO and KEGG enrichment analysis

We conducted GO and KEGG enrichment analyses using R packages including org. Hs.eg.db, colorspace, string, DOSE, clusterProfile, pathview, ggplot2, and limma. The thresholds were set at pvalueCutoff = 0.05 and qvalueCutoff = 0.05.

GO functional analysis includes three parts: Biological Process (BP), Cellular Component, and Molecular Function (MF). We selected the top 10 entries with P < 0.05 for visualization. The size of the circles or the length of the lines represents the number of genes enriched in GO/KEGG; color represents the significance of the enrichment.

### 2.8 Machine learning

We applied the “caret” package in R to build machine learning models, including Random Forest (RF), Support Vector Machine (SVM), Generalized Linear Model (GLM), and Extreme Gradient Boosting (XGB). We evaluated the reason for model establishment using residuals and ROC curves and identified the top 10 variables as central genes.

### 2.9 Differential expression and ROC analysis

To validate the accuracy of each identified hub gene, receiver operating characteristic (ROC) curve analysis was performed. Genes with an area under the curve (AUC) greater than 0.7 were considered useful for disease diagnosis. Boxplots, generated using the “ggplot2” package in R, displayed the expression levels of these key genes in UC samples compared to control samples.

### 2.10 Molecular docking simulation

The three-dimensional (3D) structures of the drugs were obtained from the PubChem database (https://pubchem.ncbi.nlm.nih.gov/). Target proteins were sourced from the RCSB Protein Data Bank (http://www.rcsb.org/). Molecular optimization was performed using SYBYL-X 2.0 software with the following parameters: Tripos force field, Gasteiger-Hückel charges, maximum iterations set to 10,000, and an energy gradient limit of 0.005 kcal/(mol·Å). All other parameters were set to their default values. Target preprocessing was carried out using mgltools_win32_1.5.6 software, and the targets were saved in PDBQT format. Molecular docking was conducted using AutoDock Vina 1.1.2 software (http://vina.scripps.edu/) to assess the affinity between the compounds and target proteins. The final graphical visualization of the docking data was accomplished using PyMOL. The evaluation criterion was a binding energy of less than −7 kcal/mol, indicating good binding between the compound and the target. Some studies require results to be presented in kJ/mol, with the conversion factor being 1 kcal = 4.184 kJ.

## 3 Materials

Experimental Animals: Forty specific pathogen-free (SPF) grade male C57BL/6 mice, aged 6–8 weeks and weighing approximately 20 g, were purchased from SpePharm Biotechnology Co., Ltd. Experimental Drugs: dextran sulfate sodium (DSS) was purchased from MP Biomedicals, United States (Catalog No.: 160110); mesalazine enteric-coated tablets were purchased from Kuihua Pharmaceutical (National Drug Approval No. H19980148). Main Experimental Reagents: AMPK was purchased from Wuhan Servicebio Technology Co., Ltd. (GB113685-50); PPARγ was purchased from Wuhan Servicebio Technology Co., Ltd. (GB11163-100); SIRT1 was purchased from Wuhan Servicebio Technology Co., Ltd. (GB11512-100); hematoxylin-eosin staining kit (Wuhan Servicebio Technology Co., Ltd., G1003).

### 3.1 Establishment, grouping, and treatment of UC animal model

A total of 40 SPF-grade male C57BL/6 mice, aged 6–8 weeks and weighing approximately 20 g, were selected. The mice were housed in the Experimental Center of Henan Provincial Hospital of Traditional Chinese Medicine. The experimental ethics number is Z-HNSZYY-2024-092.

The 40 mice were randomly divided into four groups using a random number table: normal group, model group, mesalazine group, and Gegen Qinlian component Gegen Qinlian nano-preparation group, with 10 mice in each group. Upon arrival, the mice were housed under controlled conditions: temperature maintained at 18°C–23°C, humidity at 40%–60%, and a 12-h light/dark cycle. After acclimating in the animal facility for 1 week, the widely - recognized DSS method can be employed to establish a mouse model of UC. Specifically, mice in the model group, mesalazine group, and Gegen Qinlian component nano-targeted TCM group were administered 3% DSS continuously for 7 days. Drug intervention started on the 4th day of modeling. The mesalazine group and the Gegen Qinlian component Gegen Qinlian nano-preparation group received mesalazine and Gegen Qinlian component Gegen Qinlian nano-preparation, respectively, via oral gavage at a dose of 100 mg/kg per day and a volume of 0.2 mL per mouse. The normal group and the model group received an equivalent volume of saline. The treatment duration was 7 days.

### 3.2 Changes in the general condition of mice

The disease activity index (DAI) score and the rate of body weight change in mice comprehensively reflect the inflammatory state of the mice and serve as crucial indicators for assessing the success of UC model preparation. The DAI was calculated at baseline and the end of the intervention based on previous literature. The formula for DAI is: DAI = (body weight loss score + fecal consistency score + bloody stool score)/3. Mouse Body Weight Recording: All mice were weighed daily to monitor body weight changes and record the data. The body weight change percentage was calculated as: Body weight change percentage = (daily body weight − initial body weight)/initial body weight × 100%.

Measurement of Colonic Length and colitis macroscopic damage index (CMDI) Assessment: Colonic length is a sensitive indicator of the inflammatory status in UC mice. With increasing inflammation, the colon exhibits edema and shortening. After drug intervention, mice were euthanized by cervical dislocation, and the colon tissue from the anus to the ileocecal junction was excised and measured for length. The CMDI was assessed for each group of mice as follows: Normal colon appearance: 0. Mild hyperemia without ulceration or erosion: 1. Mild hyperemia with ulceration and erosion: 2. Single ulceration or erosion with a longitudinal diameter ≤1 cm: 3. Multiple ulcerations or erosions with a longitudinal diameter >1 cm: 4.

#### 3.2.1 Spleen index

The spleen is a central immune organ and inflammatory responses often lead to an increase in spleen weight. The spleen index was calculated as: Spleen index = (spleen weight/mouse body weight) × 10.

### 3.3 Hematoxylin - eosin staining for observation of histopathological changes

Following drug intervention, mice were euthanized by cervical dislocation, and colon, lung, and spleen tissues were excised, fixed in 4% formaldehyde for more than 24 h, trimmed, dehydrated with ethanol, embedded in paraffin, and sectioned at approximately 4 μm using a microtome. The sections were placed in a water bath at about 40°C, mounted on glass slides, dried thoroughly, deparaffinized with xylene (I, II, and III) for 10 min each, and washed with descending ethanol concentrations and distilled water for about 2 min. Hematoxylin-eosin staining was performed, followed by washing and mounting. The sections were observed under an inverted microscope to evaluate histopathological and morphological changes in the tissues (Jang et al., 2019).

### 3.4 Immunohistochemical (IHC) analysis

Paraffin-embedded colon tissue sections were deparaffinized with xylene, hydrated with gradient ethanol concentrations, and subjected to heat-mediated antigen retrieval using an antigen retrieval solution. After cooling, the sections were washed with PBS three times, each for about 5 min, and incubated in 3% hydrogen peroxide at room temperature, protected from light, for 10 min, followed by three PBS washes, each for 3 min. The sections were blocked with 5% bovine serum albumin (BSA), and primary antibodies (1:1,000 dilution) against AMPK, PPARγ(1:200 dilution), and SIRT1 (1:400 dilution) were applied and incubated overnight at 4°C. After three PBS washes, the sections were incubated with secondary antibodies for 15 min at room temperature. Following three PBS washes, the sections were stained with DAB, counterstained with hematoxylin, washed until blue, dehydrated, and mounted. Microscopic observation revealed brownish-yellow granules as positive protein expression, and quantitative analysis was performed using ImageJ software.

### 3.5 Statistical analysis

Data were statistically analyzed using GraphPad Prism 9.0 software. Paired t-tests were used for comparisons between two groups, while one-way ANOVA was applied for comparisons between multiple groups when variances were equal, or Welch’s ANOVA was used when variances were unequal. A P-value < 0.05 was considered statistically significant.

## 4 Results

### 4.1 Acquisition of macrophage M2-related genes in UC patients treated with GGQL

**FIGURE 1:**
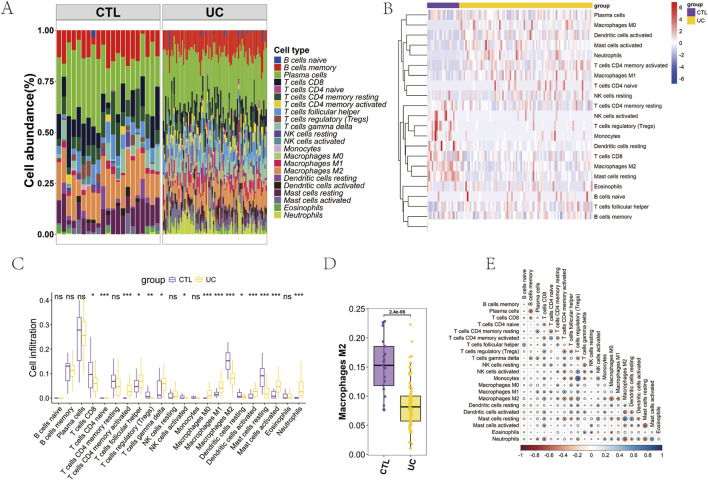
CIBERSORT Immune Infiltration. **(A)** Cumulative histogram heatmap of 22 immune cell types; **(B)** Expression heatmap of 22 immune cell types; **(C)** Differential expression bar chart of 22 immune cell types; **(D)** Differential expression histogram of macrophage M2; **(E)** Correlation heatmap of 22 immune cell types.

Microarray data (GSE92415) was downloaded from the GEO database using the search terms “Ulcerative Colitis” and “*H. sapiens*.” This comprehensive dataset includes information from 87 UC patients and 21 healthy controls. An immune infiltration analysis was conducted utilizing the CIBERSORT algorithm. The analysis indicated that Plasma cells and memory B cells comprised a significant portion of the samples (refer to Figure 1A). All immune cells and their functions, barring activated NK cells, were present in the samples (see Figure 1B). Notably, distinct subtypes of macrophages exhibited varied expression patterns in the UC samples (Figure 1C). Specifically, macrophage M0 and M1 subtypes were significantly increased, while the M2 subtype was markedly reduced in the UC cohort (refer to Figure 1D). Following this, a correlation analysis was performed across 22 distinct immune cell types. The findings revealed a significant negative correlation between Macrophage M2 and several cell types, including CD8 T cells, resting CD4 memory T cells, Tregs, and Monocytes. Conversely, a positive correlation was observed between Macrophage M2 and activated CD4 memory T cells, Thf cells, as well as Macrophage M0 and M1 subtypes (Figure 1E).

**FIGURE 2:**
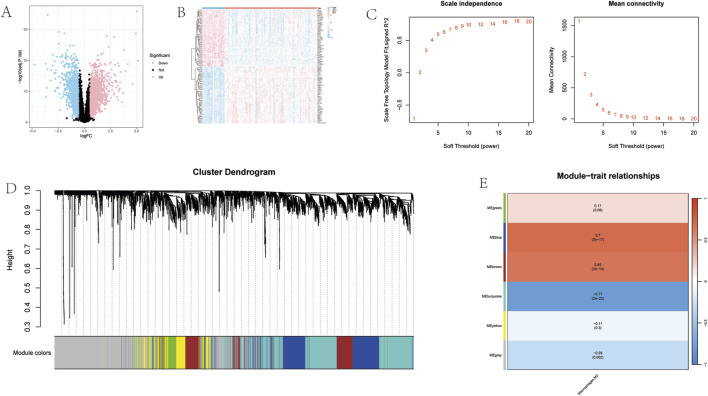
Acquisition of M2 Macrophage Targets for GGQL Treatment of UC. **(A)** Volcano plot of UC differentially expressed genes (DEGs); **(B)** Heatmap of UC DEGs; **(C)** WGCNA soft-thresholding plot’. **(D)** WGCNA gene dendrogram; **(E)** WGCNA gene heatmap.

UC-DEGs were identified using the “limma” R package, applying the thresholds of |Log_2_fold change| > 0.5 and adj. P < 0.05. This analysis identified a total of 3011 UC-DEGs, including 1676 upregulated and 1335 downregulated genes (Figures 2A, B). To ensure data integrity, hierarchical clustering was performed on the study samples based on the M2 macrophage immune infiltration phenotype, enabling the detection and removal of outliers. A scale-free network was subsequently constructed, with the soft-thresholding power β = 14 using the pickSoftThreshold function (scale-free *R* ^2^ = 0.798) (Figure 2C). Following this, an adjacency matrix was generated and converted into a Topological Overlap Matrix (TOM). Gene dendrograms and module colors were then derived based on varying degrees of connectivity (Figure 2D). Notably, the MEblue, MEbrown, and MEturquoise modules exhibited significant correlations with M2 macrophages, with correlation coefficients of 0.7, 0.65, and −0.77, respectively (Figure 2E).

### 4.2 Construction and topological analysis of the protein-protein interaction (PPI) network

**FIGURE 3:**
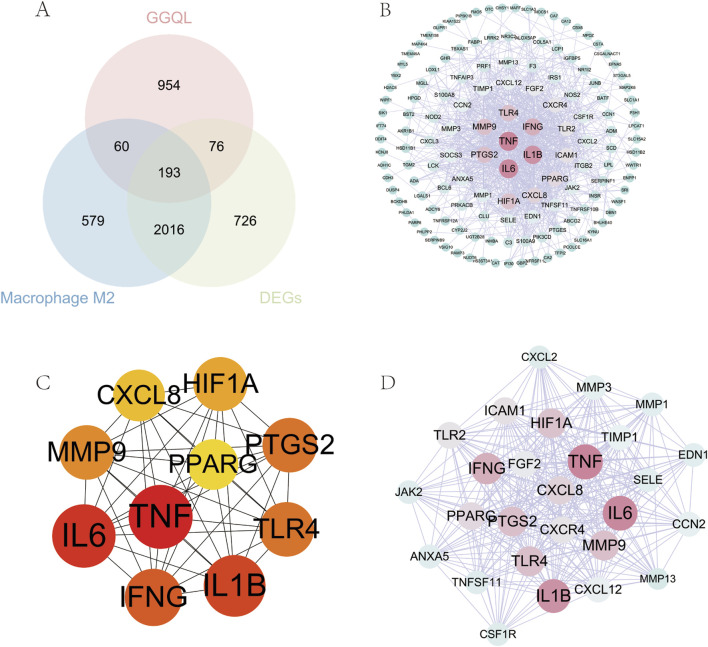
Topological Analysis of Protein Interaction Networks. **(A)** Venn diagram of M2 macrophage targets for GGQL treatment of UC; **(B)** Construction of protein interaction networks; **(C)** Core target network diagram (MCC algorithm); **(D)** Module 1 of MCODE protein clustering analysis.

Target prediction using SwissTargetPrediction, BATMAN 2.0, and the Herb Component Target Database identified 1,283 potential targets. The intersection of these datasets revealed 193 M2 macrophage targets associated with GGQL treatment for UC (Figure 3A). The intersection target dataset was imported into the STRING database (https://string-db.org/), with the species specified as *H. sapiens* and the minimum required interaction score set to 0.4. The resulting “string_interactions_short.tsv” file was downloaded and imported into Cytoscape 3.8.2 for network visualization. Analysis of the PPI network revealed that TNF, IL6, IL1B, and IFNG exhibited the highest degree values (Figure 3B). To identify core targets, the Cytohubba plugin was utilized with four distinct algorithms. The Maximum Neighborhood Component (MNC) algorithm identified IL6, TNF, IL1B, IFNG, CXCL8, ICAM1, MMP9, TLR4, PTGS2, and HIF1A as core targets (Figure 3C). Furthermore, cluster analysis of the PPI network was performed using the MCODE plugin, which revealed two distinct modules. Module 1, with a score of 24, comprised 27 nodes and 312 interactions, with TIMP1, SELE, EDN1, CCN2, CXCL2, and MMP1 as the central targets (Figure 3D).

The AMPK-PPARγ pathway plays an important role in suppressing inflammation in UC, and it can inhibit the production of its core targets through multiple signaling pathways, such as AMPK blocking NF-κB nuclear translocation by phosphorylating IKKβ, or PPARγ competitively binding to NF-κB coactivators (e.g., CBP/p300), which inhibits inflammatory gene transcription and the release of pro-inflammatory factors such as TNF-α and IL-6 (Yang et al., 2025); it can also inhibit JAK1-STAT3 phosphorylation through pathways such as binding to STAT1, thereby inhibiting transcription of factors such as IL-6 and IFNG (Yang et al., 2024). Conversely, core targets such as IL6, TNF, IL1B, IFNG, etc., can also feedback regulate the AMPK-PPARγ pathway through these signaling pathways, or by exacerbating oxidative stress, etc., (Jin et al., 2025).

### 4.3 GO and KEGG enrichment analysis

**FIGURE 4:**
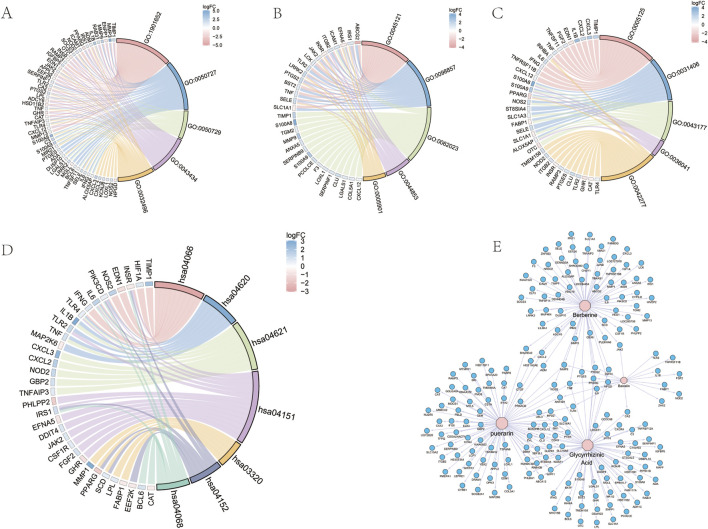
GO and KEGG functional enrichment Analysis. **(A)** GO enrichment analysis (biological Process). **(B)** GO enrichment analysis (cellular Component). **(C)** GO enrichment analysis (molecular Function). **(D)** KEGG enrichment Analysis. **(E)** Medicinal material-component-target-disease network.

GO functional enrichment analysis revealed that the primary biological processes (BPs) enriched in UC following GGQL intervention include response to peptide, regulation of inflammatory response, positive regulation of inflammatory response, response to peptide hormone, and response to lipopolysaccharide (Figure 4A). The enriched cellular components (CCs) comprise a membrane raft, membrane microdomain, collagen-containing extracellular matrix, plasma membrane raft, and caveola (Figure 4B). The main molecular functions (MFs) enriched include cytokine activity, carboxylic acid binding, organic acid binding, long-chain fatty acid binding, and peptide binding (Figure 4C). KEGG enrichment analysis indicated that GGQL treatment of UC primarily involves the AMPK-PPAR signaling pathway (Figure 4D). The herb-component-target-disease network illustrates that four components—berberine, puerarin, baicalin, and glycyrrhizin—act on multiple targets (Figure 4E).

### 4.4 Identification of key genes

**FIGURE 5:**
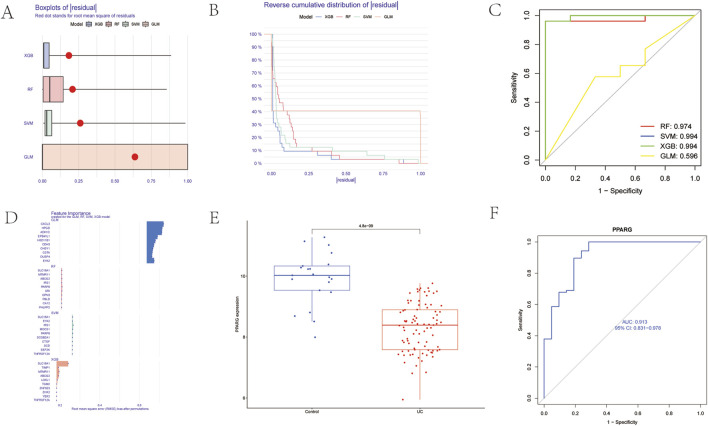
Identification of key Genes. **(A)** Machine learning residual Evaluation. **(B)** Machine learning residual Evaluation. **(C)** Machine learning ROC Evaluation. **(D)** Key gene Identification. **(E)** Key gene differential Analysis. **(F)** Key gene ROC analysis.

Based on the expression matrix of 24 genes, machine learning models were developed using the R package “caret.” Residual analysis and ROC curves were employed to assess the validity of model establishment. The results indicated that except for GLM, the residuals of the other three machine-learning methods were minimal (Figures 5A, B). Additionally, the AUC values of the ROC evaluations for these three machine learning methods exceeded 0.9, suggesting that RF, SVM, and XGB machine learning methods could all be utilized (Figure 5C). The top 10 variables were identified as central genes (Figure 5D). PPARG exhibited an AUC of 0.913, indicating that this gene possesses diagnostic value (Figure 5F), and it was significantly underexpressed in UC samples (Figure 5E).

**FIGURE 6:**
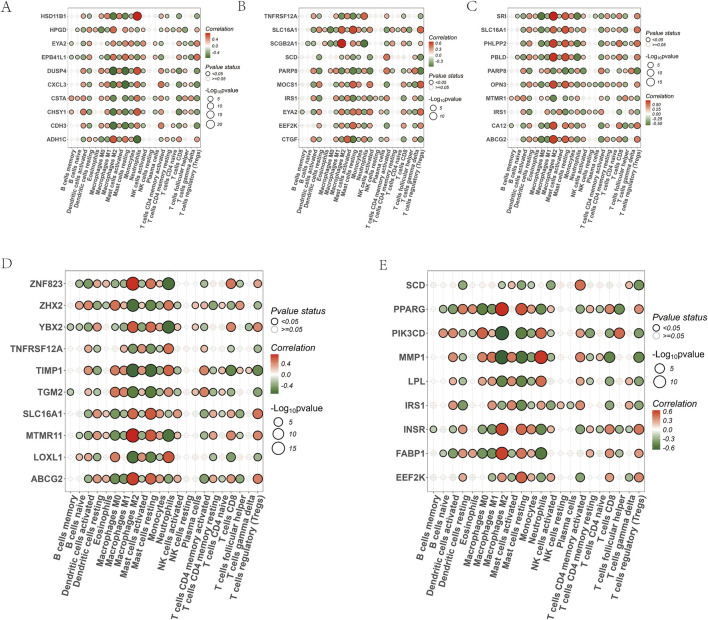
Analysis of immune correlations of core target points. **(A)** Correlation between GLM machine learning core target points and 22 immune cells; **(B)** Correlation between RF machine learning core target points and 22 immune cells; **(C)** Correlation between SVM machine learning core target points and 22 immune cells; **(D)** Correlation between XGB machine learning core target points and 22 immune cells; **(E)** Correlation between PPAR-AMPK pathway genes and 22 immune cells.

Figure 6 partially displays the immune-related analysis of key genes identified through machine learning and genes included in the AMPK-PPAR pathway. The aim is to observe the significant up-or-down-regulation of genes across 22 types of immune cells. As an example, PPARG shows a significant positive correlation with M2 macrophages (take 6E as an example).

### 4.5 Effects of Pueraria-Scutellaria nanotargeted TCM on the general condition of mice

**FIGURE 7:**
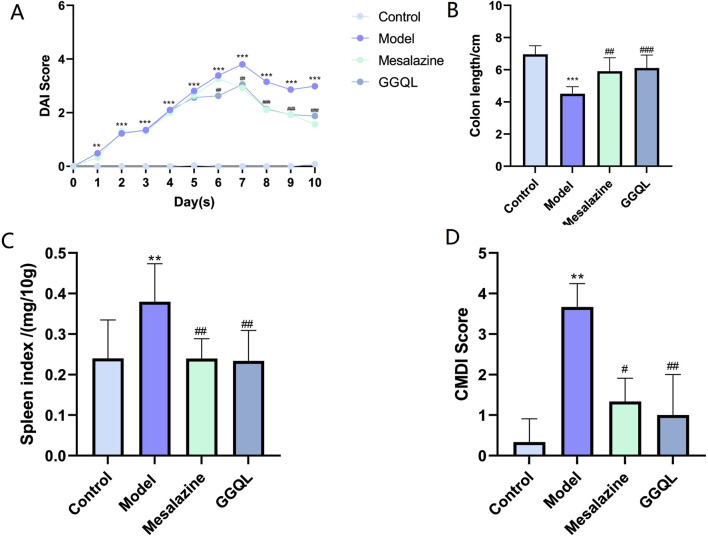
Effects of Gegen Qinlian Component Nanoparticle-Targeted TCM on General Conditions of Mice. **(A)** DAI scores in mice; **(B)** Colon length; **(C)** Spleen index; **(D)** CMDI Score. Data are represented as mean ± SD. **P < 0.01, ***P < 0.001, compared with control group; ^#^P < 0.05, ^##^P < 0.01, ^###^P < 0.001 compared with model group.

In the normal group, mice displayed good health status, high activity levels, and normal stool consistency and coloration. In the model group, after 7 days of modeling, mice exhibited lethargy, reduced activity, and symptoms of diarrhea and melena. Following treatment, mice in the Pueraria-Scutellaria nanotargeted TCM group showed significant improvements in their general condition, activity levels, fur quality, and stool consistency. Upon the free intake of 3% DSS, the DAI scores of the mice progressively increased. After 7 days of treatment, the DAI scores were significantly elevated in the model group compared to the normal group (P < 0.001). In contrast, the DAI scores of the mesalazine group and the Pueraria-Scutellaria nanotargeted TCM group were significantly reduced compared to the model group (P < 0.01) (Figure 7A).

Following 7 days of treatment, the colon length in the model group was significantly shortened compared to the normal group (P < 0.001). In comparison, the colon length in both the mesalazine group and the Pueraria-Scutellaria nanotargeted TCM group was significantly increased relative to the model group (P < 0.01) (Figure 7B).

In comparison to the normal group, the spleen index in the model group was significantly elevated (P < 0.05). Conversely, the spleen index in both the mesalazine group and the berberine group was significantly reduced compared to the model group (P < 0.05, P < 0.01) (Figure 7C).

Compared to the normal group, the CMDI scores in the model group were significantly elevated (P < 0.01). In contrast, the spleen index in both the mesalazine group and the GGQL group was significantly reduced compared to the model group (P < 0.01) (Figure 7D).

### 4.6 Effects of GGQL nano-preparation on pathological conditions of the colon, lung, and spleen in mice

**FIGURE 8:**
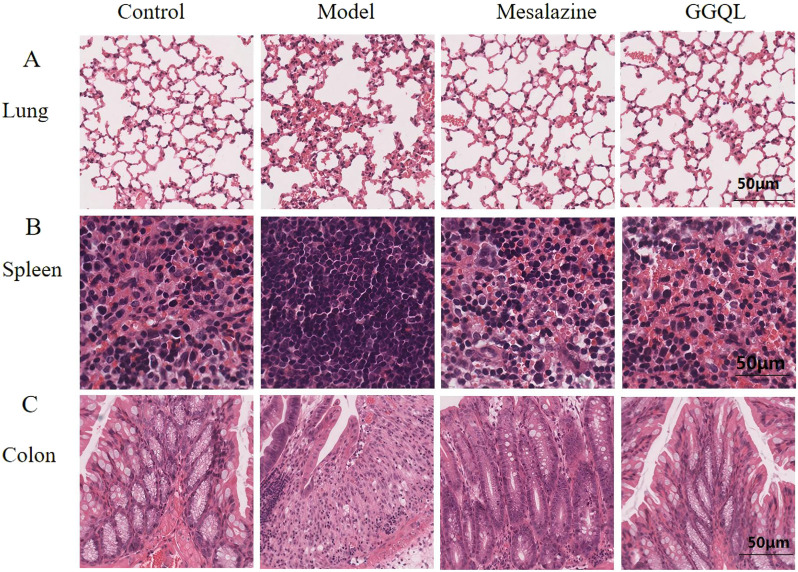
Effects of Gegen Qinlian component nanoparticle-targeted TCM on pathological conditions of the lung, spleen and colon in mice. **(A)** Effects on lung pathology; **(B)** Effects on spleen pathology; **(C)** Effects on colon pathology.

Histological examination using H&E staining revealed the following: Control Group: In the lung tissue, the structure was intact and clearly defined, with no evidence of inflammatory cell infiltration or alveolar wall thickening. Model Group: The lung tissue showed marked congestion, hemorrhage, and edema within the alveoli, accompanied by significantly thickened, ruptured, and even collapsed alveolar walls. Treatment Groups (Mesalazine and GGQL): After treatment, the alveolar structure improved significantly compared to the model group (Figure 8A). The spleen consists of two primary components: Red Pulp: Responsible for storing and releasing red blood cells to maintain blood homeostasis. White Pulp: A key component of the immune system. The distribution of red and white pulp can reflect the inflammatory status of the body. Control Group: The boundary between the red pulp and white pulp was clearly defined. Model Group: The red and white pulp regions were fused, with unclear boundaries. Treatment Groups (Mesalazine and GGQL): After treatment, the red and white pulp contours returned to a normal state, indicating an improvement in the inflammatory condition in the mice (Figure 8B). For the intestinal mucosa: Control Group: The mucosa was intact, with clear crypt contours and well-organized gland arrangement, and no neutrophil infiltration. Model Group: The mucosa was damaged, with deformed and severely disrupted crypts, some of which were absent, and disorganized gland arrangement, accompanied by obvious neutrophil infiltration. Treatment Group (Gegen Qinlian Component Nanoparticle-Targeted TCM): The intestinal mucosa and crypt structure of the mice returned to a state similar to the normal group, with efficacy comparable to that of the mesalazine group (Figure 8C).

### 4.7 GGQL nano-preparation promotes macrophage M2 polarization via the AMPK - PPARγ axis

**FIGURE 9:**
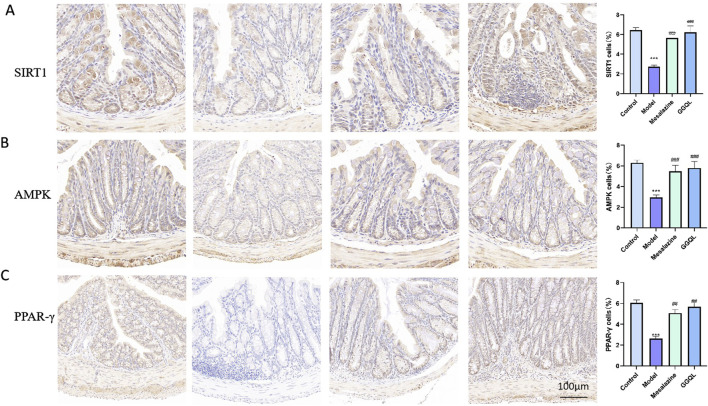
Effects of Gegen Qinlian Component Nano-Targeted TCM on Proteins Related to the AMPK-PPARγ Axis in Mouse Colonic Tissue. **(A)** Effect of Gegen Qinlian Component Nano-Targeted TCM on SIRT1; **(B)** Effect of Gegen Qinlian Component Nano-Targeted TCM on AMPK; **(C)** Effect of Gegen Qinlian Component Nano-Targeted TCM on PPARγ Data are represented as mean ± SD. ***P < 0.001, compared with control group; ^##^P < 0.01, ^###^P < 0.001 compared with model group.

Immunohistochemical results showed that, compared to the model group, the expression levels of SIRT1, AMPK, and PPAR-γ were significantly increased in the GGQL group and the mesalazine group (P < 0.01) (Figures 9A–C).

## 5 Discussion

UC is characterized by recurrent episodes and is difficult to cure, with a certain risk of malignant transformation, posing a significant threat to patient health and life (Krugliak et al., 2022). Clinically, UC is primarily manifested by persistent abdominal pain, diarrhea, mucus in the stool, and bloody diarrhea. DSS is a commonly used agent for inducing UC in mouse models (Li et al., 2022). DSS can disrupt the structure of the intestinal epithelial cells in mice, making it easier for bacteria and toxins to enter the intestine, leading to extensive inflammatory infiltration and further causing intestinal tissue edema, erosion, and bleeding (Jialing et al., 2020). In this study, the 3% DSS-induced mouse colitis model demonstrated symptoms such as diarrhea, bloody stools, weight loss, and colon shortening, which closely resemble the clinical manifestations of UC, thereby confirming the successful establishment of the animal UC model. Within this model, it was observed that the Gegen Qinlian component nano-targeted TCM effectively reduced the DAI score, mitigated the rate of weight loss, and ameliorated colon shortening. Furthermore, the crypt architecture of the colon was restored, abscesses resolved, and the red and white pulp of the spleen returned to a state comparable to that of normal mice. These results suggest that the Gegen Qinlian component nano-targeted TCM exerts a significant therapeutic effect on UC. Consequently, further investigation into the underlying mechanisms by which this TCM formulation alleviates UC is warranted.

The pathogenesis of UC results from a multifaceted interplay of factors, including damage to the mucosal mechanical, biological, immune, and chemical barriers, dysbiosis of gut microbiota, and excessive apoptosis of intestinal epithelial cells (Liu et al., 2022). In recent years, increasing attention has been paid to the dysregulation of immune responses and damage to the immune barrier in UC. Research has highlighted that pro-inflammatory macrophage polarization is a key driver of UC inflammation. Promoting the transformation of macrophages from a pro-inflammatory M1 phenotype to an anti-inflammatory M2 phenotype is a critical strategy for treating UC (Hegarty et al., 2023). Active components of TCM and TCM compound formulas activate immune cells and autophagy responses, regulate inflammatory cytokine levels, effectively modulate intestinal mucosal immune responses, promote the repair and establishment of intestinal mucosal barriers, and maintain gut microbiota homeostasis. Compared to traditional treatment regimens, TCM has fewer adverse effects (Long et al., 2022). Gegen Qinlian nano-preparation, a classical formula from Zhang Zhongjing’s “Treatise on Febrile Diseases” used for treating damp-heat diarrhea, has demonstrated a good therapeutic effect on UC. Studies indicate that Gegen Qinlian nano-preparation can maintain the dynamic balance of colonic Th17/Treg cells, effectively restoring immune homeostasis and significantly alleviating DSS-induced UC (Zhao et al., 2021; Gomez-Bris et al., 2023). Zhao et al. (2020) found that Gegen Qinlian nano-preparation enhances the low-activity Notch signaling pathway in chronic UC mice, thereby improving UC inflammatory responses. Lin et al. (2022) suggested that Gegen Qinlian nano-preparation might treat UC by activating the Nrf2 pathway to counteract oxidative stress-induced damage. Additional studies have reported (Hu et al., 2022; Li et al., 2021) that Gegen Qinlian nano-preparation can prevent the dysregulated proliferation of pathogenic Escherichia-Shigella and increase beneficial bacteria like Akkermansia and Romboutsia, thus regulating the UC gut microbiota. With the progression of contemporary science and technology, the function of TCM has transcended its conventional use in decoctions or isolated active compounds. The advent of nanotechnology-targeted drug delivery systems facilitates a more sophisticated integration of TCM with modern nanotechnological approaches. Nanomaterials are employed as carriers to encapsulate or adsorb the active constituents of TCM, thereby enhancing their efficacy and application (Contera et al., 2020). It can increase the specific surface area by reducing the particle size, promoting the active ingredients in the drug to penetrate the biofilm, and enhancing the dissolution and intestinal absorption (Xu et al., 2018); the surface of nano-herbal formulations can be modified to facilitate active targeting and enable penetration of the blood-brain barrier. Additionally, their unique shell structure can protect the active ingredient from enzymatic degradation, thereby extending the drug’s half-life and reducing the frequency of administration. Certain nano-formulations can also minimize clearance by the reticuloendothelial system through the optimization of particle size and surface charge, thereby prolonging circulation time. Consequently, nano-herbal formulations hold significant potential for clinical application when combined with existing biological agents. They may offer multi-targeted therapeutic effects that complement the highly specific mechanisms of biological agents. The integration of these two approaches could enhance therapeutic efficacy while reducing the required dosage and associated side effects of individual drugs (Huang et al., 2024). Therefore, our team has combined modern scientific techniques to prepare a nanotechnology-targeted Chinese medicine formulation based on Gegen-Qinlian components to explore its mechanism of action in treating UC.

Through comprehensive analysis, we identified the targets of GGQL’s action on macrophage M2 in UC samples, which include CXCL2, MMP1, IL6, and HIF1A, among others. GO and KEGG enrichment analyses revealed that the main active constituents of GGQL are berberine, puerarin, baicalin, and glycyrrhizin. These components can either directly regulate the AMPK-PPAR pathway, e.g., berberine and puerarin can directly promote AMPK phosphorylation, or indirectly activate the AMPK-PPAR pathway by regulating its upstream related factors, e.g., berberine induces AMPKα (Thr172) phosphorylation through activation of the upstream kinase of AMPK, LKB1, and thus induces the activation of the AMPK-PPAR pathway. The activation of AMPK-PPAR pathway is induced by the activation of AMPK upstream kinase LKB1, which induces the phosphorylation of AMPKα (Thr172), thus inducing the activation of AMPK-PPAR pathway (Rong et al., 2025; Huang et al., 2022). Baicalin inhibits the production of inflammatory cytokines such as TNF-α and IL-6, activates the PPAR signaling pathway, and exerts anti-inflammatory effects (Liao et al., 2021).

Key gene identification and immune correlation analysis confirmed that PPARG, a pivotal gene in the AMPK-PPAR pathway, is significantly downregulated in UC samples and positively correlated with macrophage M2. Subsequent experimental results indicate that Gegen Qinlian nano-preparation containing Gegen Qinlian components effectively enhances the expression of AMPK and PPARγ, as well as the expression of M2 macrophage markers such as CD163, CD20, Arg1, and F480. In summary, Gegen Qinlian nano-preparation incorporating Gegen Qinlian components promotes the polarization of M2 macrophages and ameliorates intestinal inflammatory responses by activating the AMPK-PPARγ axis.

This study, however, is subject to certain limitations. It primarily relies on animal models, indicating a need for future research to incorporate *in vitro* experiments to further elucidate the underlying mechanisms. Furthermore, clinical trials are necessary to validate the safety and efficacy of the Gegen Qinlian nano-preparation, which contains Gegen Qinlian components, in patients with UC. Considering that the pathogenesis of UC is multifactorial and involves multiple pathways, it is imperative to conduct further research to investigate the interactions between the AMPK-PPARγ axis and other signaling pathways, such as NF-κB and the NLRP3 inflammasome, and to determine their specific roles in the pathogenesis of UC.

## 6 Conclusion

Following the successful development of an animal model for UC, this study employed a Gegen Qinlian nano-preparation, consisting of Gegen Qinlian components, as a therapeutic intervention. The research findings demonstrate that nano-targeted administration of Gegen Qinlian components significantly reduces DAI scores and mitigates weight loss, while also alleviating colon length shortening. Furthermore, the study observed a restoration of colonic crypt architecture, resolution of abscesses, and normalization of the spleen’s red and white pulp, approximating the conditions observed in healthy mice. Mechanistically, treatment with nano-targeted Gegen Qinlian components markedly upregulates the expression of AMPK and PPARγ, as well as M2 macrophage markers such as CD163, CD20, Arg1, and F480. In conclusion, the Gegen Qinlian nano-preparation, comprising Gegen Qinlian components, facilitates M2 macrophage polarization and ameliorates intestinal inflammation through the activation of the AMPK-PPARγ axis.

## Data Availability

The datasets presented in this study can be found in online repositories. The names of the repository/repositories and accession number(s) can be found in the article/supplementary material.
